# Supplementary material for: Cellular preservation of musculoskeletal specializations in the Cretaceous bird Confuciusornis
Source: Nat Commun. 2017 Mar 22;8:14779. doi: 10.1038/ncomms14779 (PMC5364438; doi:10.1038/ncomms14779)
Supplement: Supplementary Information — Supplementary Figures, Supplementary Table and Supplementary References [file ncomms14779-s1.pdf]

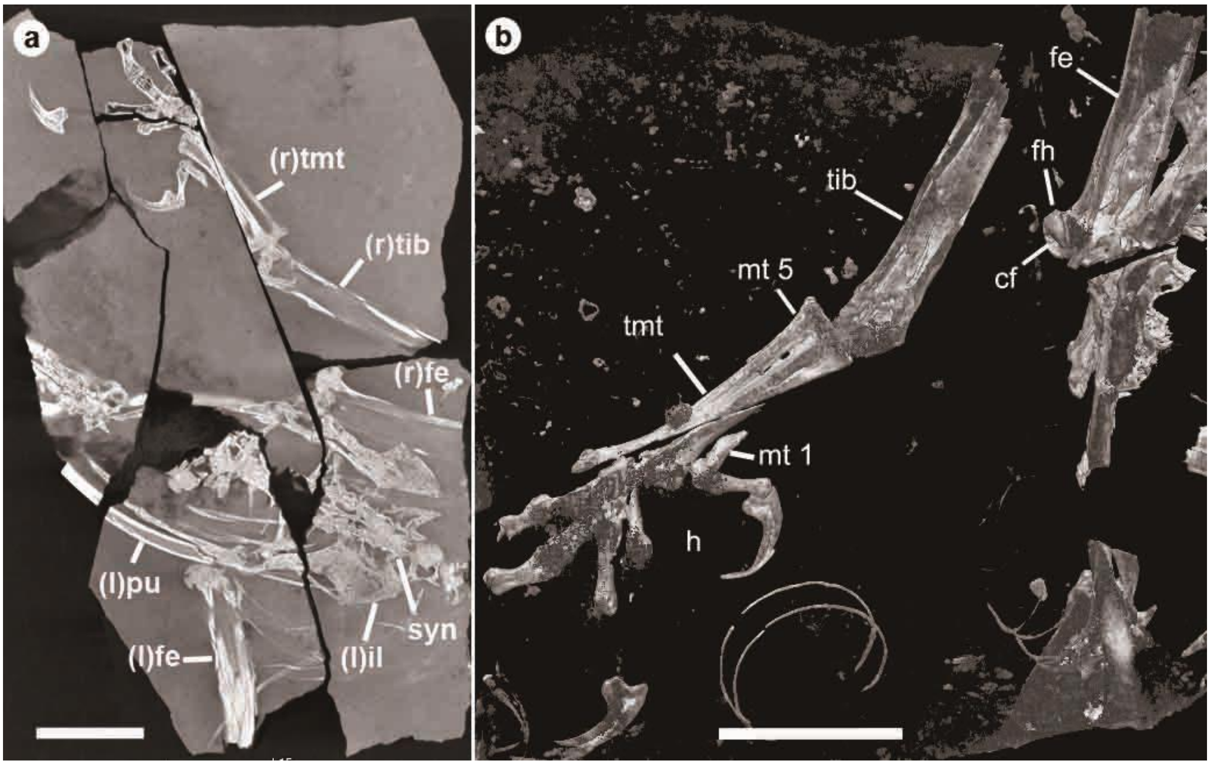

Supplementary Figure 1. CT scan image of *Confuciusornis* specimen MES-NJU 57002 (a) and a dorsomedial view of the right lower limb (b). Abbreviations: cf, capital fossa; fe, femur; fh, femoral head; h, hallux; il, ilium; mt 1, metatarsal I; mt 5, metatarsal V; pu, pubis; syn, synsacrum; tib, tibiotarsus; tmt, tarsometatarsus; l/r, left/right. Scale bars: 15 mm.

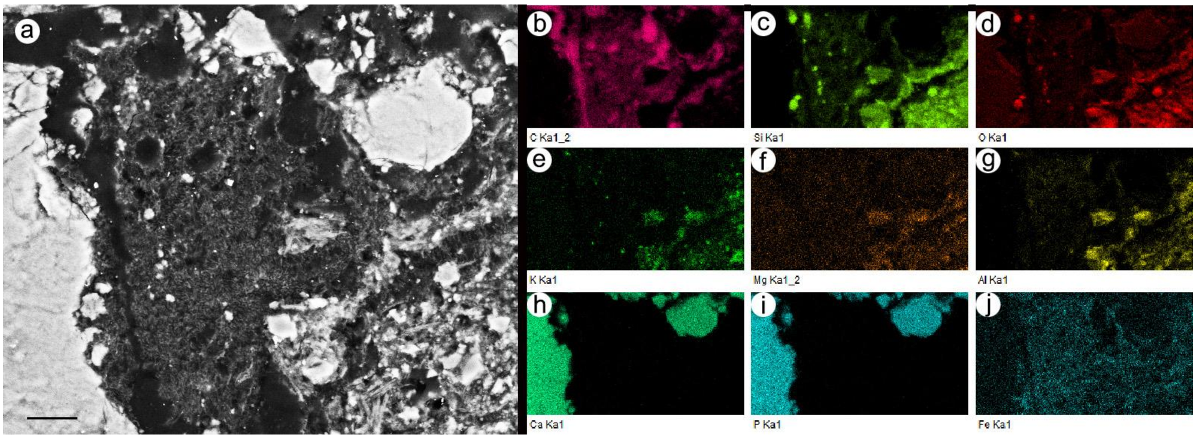

Supplementary Figure 2. BSEM image (**a**) and BSEM-based Energy dispersive X-ray element mapping (**b-j**) show that the soft tissues comprise mainly carbonaceous materials. Scale bar in **a** is 10 µm.

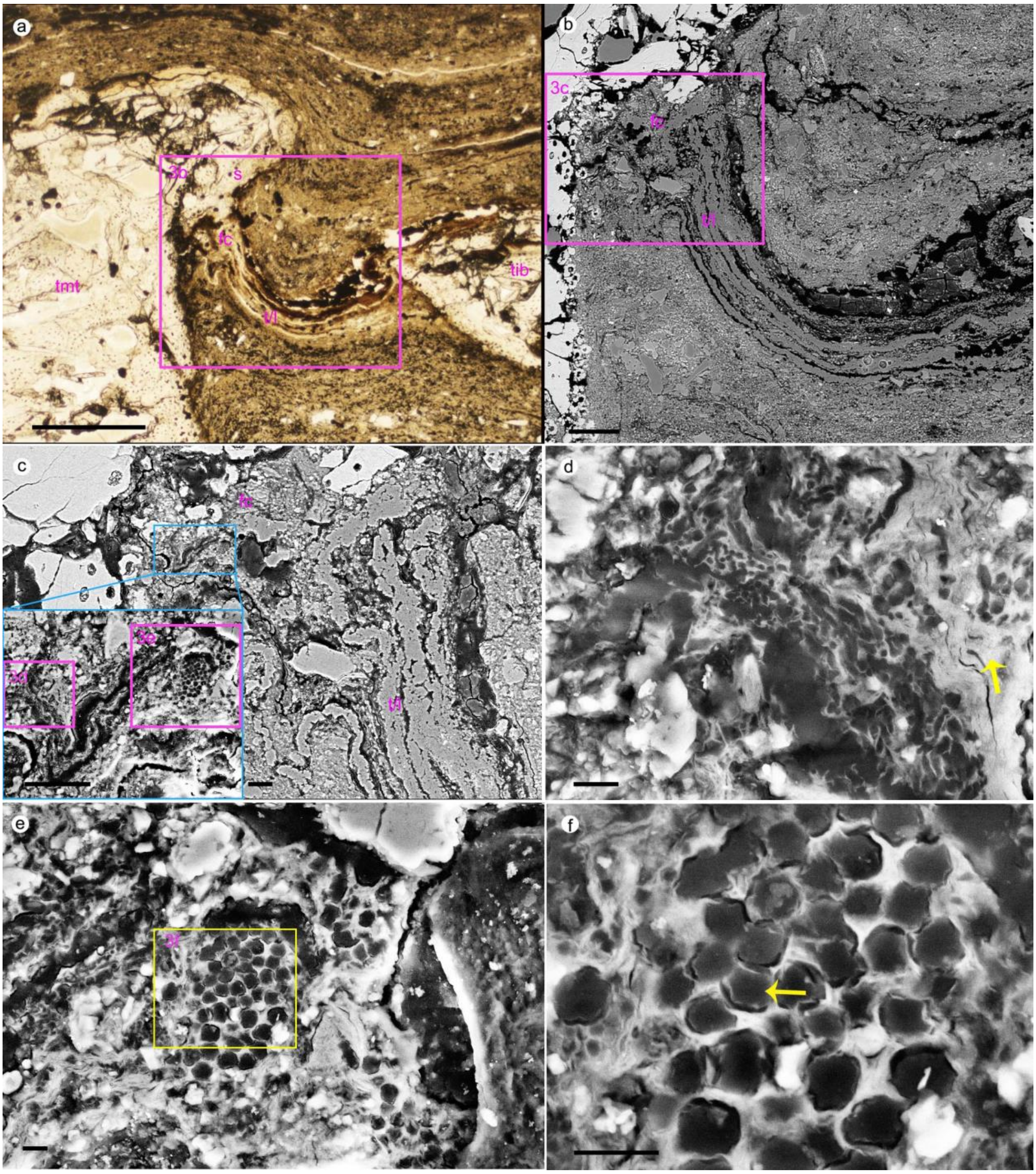

Supplementary Figure 3. Morphology of putative tendons/ligaments and fibrocartilage in the *Confuciusornis* specimen. (**a**) Photomicrograph. (**b**) BSEM image of the square area in **a**. (c) Close-up view of the square area in **b**. (**d**) Close-up view of the square area in the inset image in **c**. (**e**) Close-up view of the another square area in the inset image in **c**. (**f**) Close-up view of the square area in **e**. Note the parallel arrangement of the fibrils forming the bundles in longitudinal (arrow in **d**) and approximate cross-sectional views (arrow in **f**) Abbreviations: see Figs. 1 and 2. Scale bar: 500μm in **a**, 200 μm in **b**, 20 μm in **c**, 2 μm in **d**-**f**.

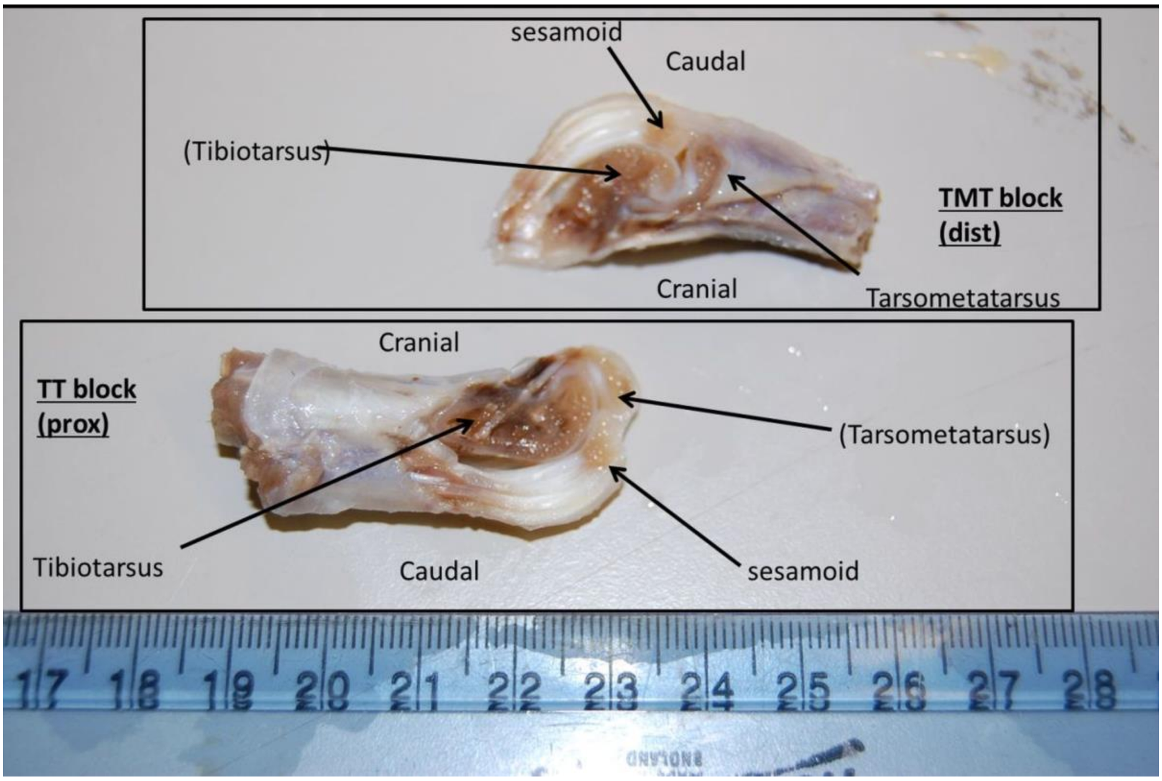

Supplementary Figure 4. Same anatomical regions in an extant bird as in the main fossil specimen. Left distal tibiotarsus (TT; below) and proximal tarsometatarsus (TMT; above) from an adult helmeted guineafowl (*Numida meleagris*) after formalin fixation. Scale bar (ruler) is in cm.

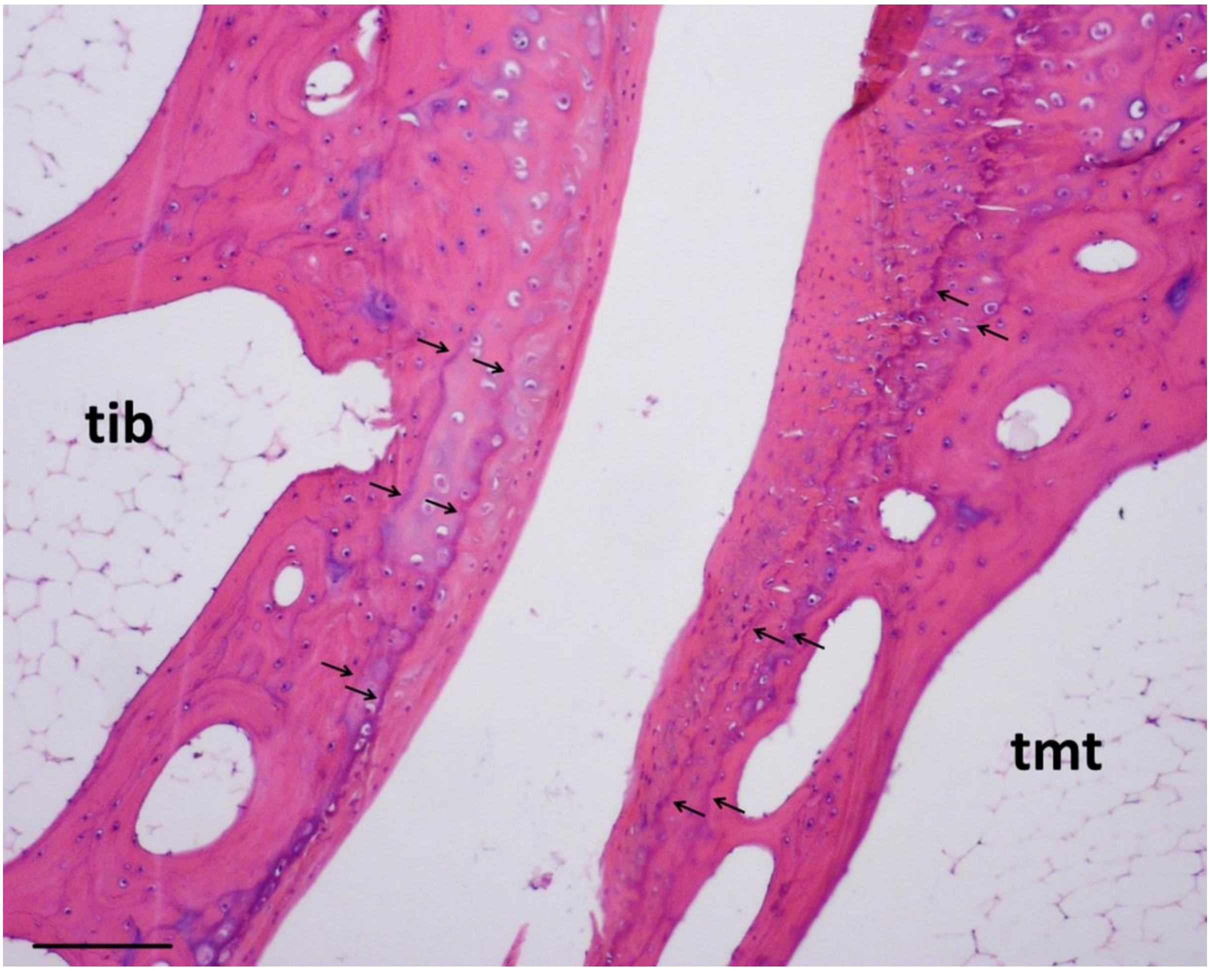

Supplementary Figure 5. H&E stained quail (*Coturnix coturnix*) ankle, showing the articular surfaces of the tibiotarsus (tib) and tarsometatarsus (tmt). Tidemarks are visible (arrows) between the bone and calcified cartilage layer, and between the calcified and uncalcified cartilage layers. Rounded chondrocyte lacunae within the articular cartilage measure approximately 10um. Scale bar is 100um.

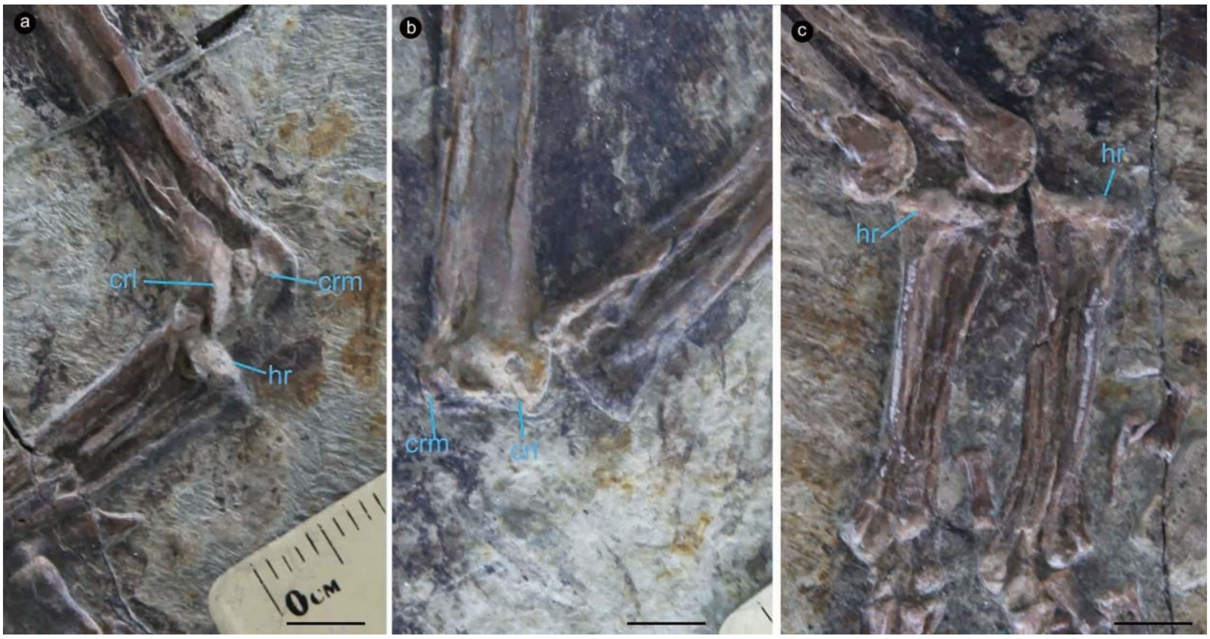

Supplementary Figure 6. Plantar views of the ankle joints in three prepared *Confuciusornis* specimens from the Institute of Vertebrate Paleontology and Paleoanthropology, Chinese Academy of Sciences (IVPP). (**a**) Left hindlimb of IVPP18156. (**b**) Right hindlimb of IVPP13175. (**c**) Both hindlimbs of IVPP13168. Abbreviations: see Figs. 1 and 2. Scale bars are 1 cm.

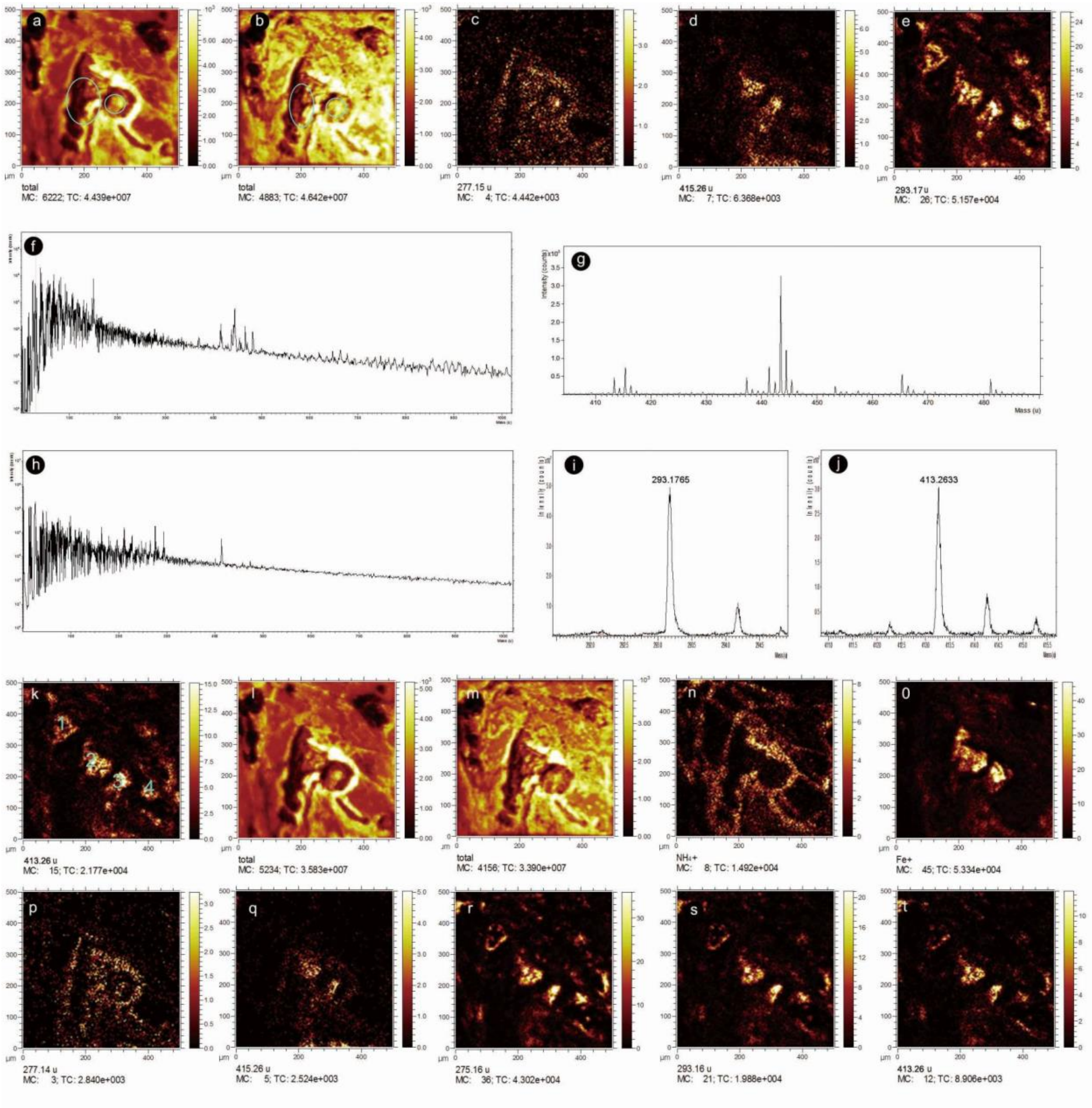

Supplementary Figure 7. Spectra and ion images from ToF-SIMS analysis. (**a-e**, **k**) Ion images of the spatial signal intensity distribution for total positive ions (**a**), total negative ions (**b**), the peaks at 277.15 amu (**c**) and 415.26 amu (**d**) in the positive spectrum, and the peaks at 293.17 amu (**e**) and 413.26 amu (**k**) in the negative spectrum. (**f-j**) Spectra obtained at analysis depth up to 1 nm from the areas enclosed by the blue line in **a** and **b**. (**f-g**) Positive spectrum (**f**) and detailed region in 410-490 amu (**g**). (**h-j**) Negative spectrum (**h**) and detailed regions in 291-295 amu (**i**) and 411-415.5 amu (**k**). (**l-t**) Ion images of the spatial signal intensity distribution acquired at analysis depth up to 1.7 nm. (**l**) Total positive ions. (**m**) Total negative ions. (**n**) NH_4^+. (**o**) Fe^+. **p-t**, The peaks at 277.14 amu (**p**) and 415.26 amu (**q**) in the positive spectrum, and the peaks at 275.16 amu (**r**), 293.16 amu (**s**) and 413.26 amu (**t**) in the negative spectrum.

Supplementary Table 1. Comparison of tarsometatarsus length of the studied *Confuciusornis* specimen with those of other known specimens.

Specimen	Tarsometatarsus length (mm)	Data source
MES-NJU 57002	21	This study
IVPP V11372	24	This study
IVPP V13156	30	Supp. Ref. 1; this study
IVPP V11619	25	Supp. Ref. 2
IVPP V10895	21	Supp. Ref. 3
IVPP V10915	32	Supp. Ref. 3
GMV-2130	23	Supp. Ref. 4
GMV-2131	21	Supp. Ref. 4
GMV-2133	26	Supp. Ref. 4

Supplementary References

1 Zhou, Z. & Zhang, F. Mesozoic birds of China-a synoptic review. *Front. Biol. China* **2**, 1–14 (2007).

2 Zhou, Z. & Zhang, F. A long-tailed, seed-eating bird from the Early Cretaceous of China. *Nature* **418**, 405–409 (2002).

3 Hou, L., Zhou, Z., Gu, Y. & Zhang, H. *Confuciusornis sanctus*, a new Late Jurassic sauriurine bird from China. *Chinese Sci. Bull.* **40**, 1545–1551 (1995).

4 Chiappe, L. M., Ji, S.-A., Ji, Q. & Norell, M. A. Anatomy and systematics of the Confuciusornithidae (Theropoda, Aves) from the late Mesozoic of northeastern China. *Bull. Am. Mus. Nat. Hist.* **242**, 1–89 (1999).
